# Herlyn Werner Wunderlich Syndrome Presenting with Ischemic Stroke due to Suspected Paroxysmal Nocturnal Hemoglobinuria: A Case Report

**DOI:** 10.31729/jnma.5838

**Published:** 2021-02-28

**Authors:** Ayushma Acharya, Prajwala Yogi, Pramod Singh, Tulsi Ram Bhattarai

**Affiliations:** 1 Kathmandu Medical College and Teaching Hospital, Sinamangal, Kathmandu, Nepal; 2 Faisalabad Medical University, Punjab, Pakistan; 3 Department of Internal Medicine, Kathmandu Medical College and Teaching Hospital, Sinamangal, Kathmandu, Nepal

**Keywords:** *cerebral ischemia*, *paroxysmal nocturnal hemoglobinuria*, *stroke*

## Abstract

Paroxysmal nocturnal hemoglobinuria can rarely present as cerebral ischemia and stroke due to arterial thrombosis. However, it should be considered in a young patient with bone marrow failure features, systemic thromboses, and hemolysis. The variants of paroxysmal nocturnal hemoglobinuria pose a diagnostic challenge and hence are important to recognize. We report a case of a 28-years-old female with Herlyn Werner Wunderlich Syndrome who presented with an ischemic cerebrovascular accident, pancytopenia, hemoglobinuria, and widespread abdominal thromboses suggestive of paroxysmal nocturnal hemoglobinuria. The patient was managed symptomatically and referred to a hematologist.

## INTRODUCTION

Paroxysmal nocturnal hemoglobinuria (PNH) is an acquired hematopoietic stem cell disorder with a triad of chronic hemolysis, bone marrow failure, and thromboses.^1^ Mutation involving Phosphatidylinositol glycan (PIG-A) gene causes failure to synthesize glycophosphatidylinositol anchor proteins, including the complement regulators CD55 and CD59, on the surface of affected blood cells, making them vulnerable to complement activation, the formation of membrane attack complex, and eventually hemolysis.^2^

PNH-AA (Aplastic Anaemia) overlaps present with lower reticulocyte count, severe thrombocytopenia, normal lactose dehydrogenase (LDH) levels, and may even evolve into myelodysplastic syndrome.^3-5^

We report a case of a 28-years-old female with Herlyn Werner Wunderlich Syndrome who presented with an ischemic cerebrovascular accident, pancytopenia, hemoglobinuria, and widespread abdominal thromboses suggestive of PNH.

## CASE REPORT

A 28 years old female presented to the emergency department on 12th April 2020 with chief complaints of slurring of speech for two days and weakness of the right half of the body. The patient woke up in the morning two days back, realizing she could not speak properly and could only produce incomprehensible sounds. The following day, the patient noticed the weakness of the right half of her body and was unable to walk or grasp things with her right hand. Her relatives noticed a left-sided deviation of her face. The patient was a normotensive, non-diabetic, non-smoker with no thyroid disorder. She was currently not under any oral or injectable contraceptives.

On examination, her vitals were within normal limits, with the Glasgow Coma Scale (GCS): eye-opening E4, verbal response V2, and motor response M6. Wrinkling of the forehead was present with left-sided facial deviation and loss of nasolabial fold, suggestive of an upper motor nerve lesion (UMN) type of facial nerve palsy. She had hypertonia of the right upper limb with reduced power on both upper and lower limbs and an upgoing plantar reflex on the same side.

The patient had been referred from another hospital where her investigations showed hemoglobin of 9.0 mg/dl, packed cell volume (PCV) 28.7, total count (TC) 2600/mm^3^, differential count (DC), neutrophil (N) 82, lymphocyte (L) 15 and platelet count of 60000/mm^3^. The patient's liver function test (LFT) showed normal liver enzymes with indirect hyperbilirubinemia. Urine routine microscopic examination showed a light red-colored urine, acidic, containing albumin and red blood cells. The urine pregnancy test was negative.

The patient was admitted to neuro medicine intensive care unit (ICU) for further management. The patient had normal fasting blood sugar, sodium, potassium, urea, creatinine, total protein, albumin, and uric acid.

**Figure 1.**
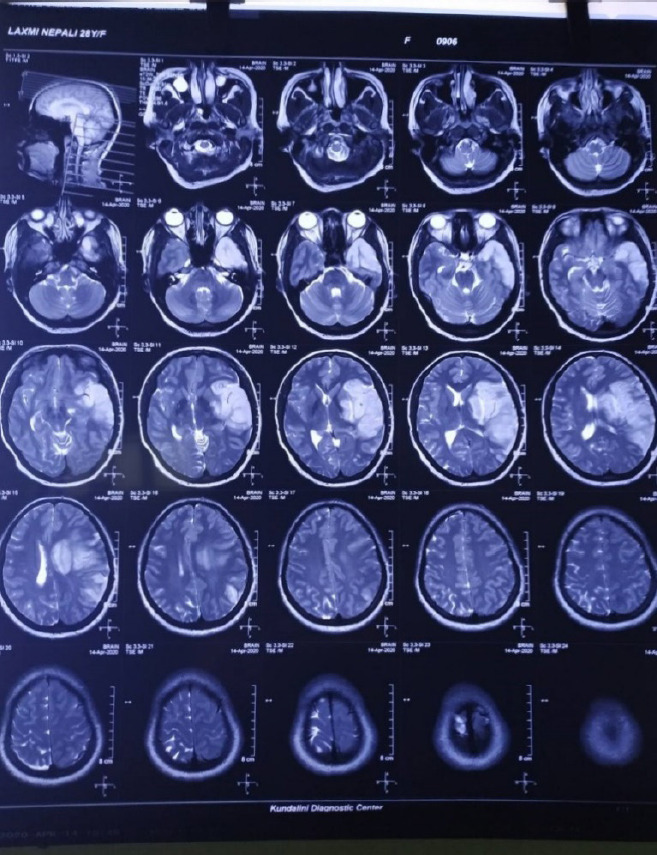
MRI showing Acute Infarct in Left Straitocapsular region along with Left Frontal and Temporal lobe i.e., the Left MCA territory.

Magnetic resonance and Imaging (MRI) of the brain showed features suggestive of acute infarct in the left straitocapsular region along with left frontal and temporal lobe i.e., the left middle cerebral artery (MCA) territory with mass effect and a midline shift (Figure 1).

A thrombus was noted in the left MCA with partial recanalization. Injection mannitol and injection frusemide were started to reduce the intracranial pressure due to the mass effect. Tablet propranolol was given to reduce the recurrence of stroke.

The patient's weakness progressively increased by 3^rd^ day with a drop in power of the right upper and lower limb from 3/5 to 1/5.

Her blood count still showed features of pancytopenia. The patient's iron profile showed features of chronic iron deficiency. Her lactose dehydrogenase (LDH) was normal at 244 U/L. Supplemental iron, folic acid, and vitamin B12 were started (Table 1).

**Table 1:** Investigations in the hospital.

Variables	Values
Total bilirubin (mg/dl)	2.3
Direct bilirubin (mg/dl)	0.3
^*^SGOT/||SGPT/†ALP	32/18/45
Protein (gm/dl)	6.9
Albumin (gm/dl)	4.1
Calcium (mg/dl)	8.8
Phosphorus (mg/dl)	3.3
‡CPK NAC (U/L) /Troponin I	69/negative
LDH (U/L)	244
Urine routine and microscopy test	Light red urine (Intially)
Color	Light yellow (Later)
Platelets count	2-3
Eosinophil count	2-3
Red Blood Cell	2-3
	plenty
Urine for Dysmorphic RBC	Not seen
Red cell Indices	
Mean corpuscular volume (fl)	71.4
Mean corpuscular hemoglobin (pg)	22.3
Mean corpuscular hemoglobin concentration (gm%)	31.2
VDRL	Non-reactive
¶HBsAG/**HIV/||||HCV	Non-reactive
Serum uric acid (mg/dl)	2.5
Serum iron (mcg/dl)	35
Total iron binding capacity	380
Serum ferritin (ng/ml)	5.42
‡‡PT/INR	18/1.3
Activated partial thromboplastin	
Test	26
Control	26
Thyroid function test	
‡‡FT3 (pg/ml)	2.58
¶¶FT4 (pg/ml)	14.32
****TSH (μIU/ml)	2.28
Vitamin B12 (pg/ml)	2000

* SGOT: Serum glutamic oxaloacetic transaminase, ||SGPT: Serum glutamic pyruvic transaminase, †ALP: Alkaline phosphatase, ‡CPK NAC: Creatine phosphokinase N-acetyl-cystein, ¶HBsAG: Hepatitis B surface antigen, **HIV: Human immunodeficiency virus, ||||HCV: Hepatitis C virus, ††PT/INR: Prothrombin Time and International Normalized Ratio, ‡‡FT3: tri-iodothyronine, ¶¶FT4: thyroxine, ***TSH: thyroid stimulating hormone.

Thyroid function test, vitamin B12 were normal, and Venereal disease research laboratory test (VDRL) for syphilis was non-reactive, ruling out these as the cause of stroke.

Peripheral blood smear showed normocytic normochromic anemia, mild anisopoikilocytosis, few microcytes, hypochromic cells, and pencil cells, along with leukopenia and thrombocytopenia. Reticulocyte count was 0.5%. The patient was consulted with a hematologist who advised for bone marrow biopsy, which the patient refused.

Echocardiography showed a normal left ventricular systolic function with ejection fraction (EF) 61%, no intracardiac mass or intracardiac shunt with normal valvular function. Ultrasonography (USG) abdomen and pelvis showed features suggestive of hemangioma of the left lobe of liver, chronic portal vein thromboses, and mild splenomegaly.

**Figure 2.**
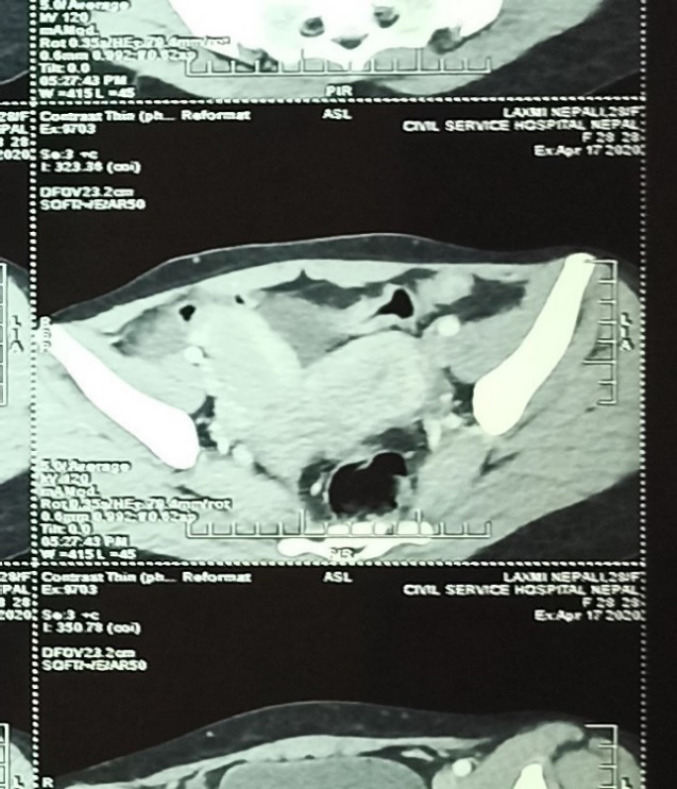
CECT abdomen showing uterine didelphys.

Contrast-enhanced computed tomography (CECT) abdomen showed extrahepatic portal vein obstruction with a feature of sequelae of portal hypertension (Figure 2).

Portal and Splenic veins were not visualized and were replaced by multiple tortuous collaterals. Spleen was enlarged, measuring 14.3 cm in craniocaudal direction. Scan also showed agenesis of the right kidney with uterine didelphys. The patient had a history of dysmenorrhea a few months before her menarche and had undergone a surgical procedure for the same after abdominal pain. Her features were suggestive of Herlyn Werner Wunderlich syndrome with a triad of renal agenesis, uterine didelphys and obstructed hemivagina diagnosed radiologically.

From day 7th to 16th patient's blood count had further deteriorated with platelets as low as 28000/mm^3^. The patient was transfused with 2 pints of platelet-rich plasma.

From day 17 patient started showing improvement with increasing power in both upper and lower limb, reduction in slurring of speech with the patient being able to produce comprehensible words, and was started on physiotherapy.

Anticardiolipin IgG and IgM, antiphospholipid IgG and IgM and Anti-nuclear antibodies were all negative ruling out autoimmune connective tissue disorder in the patient (Table 2). The patient had raised an erythrocyte sedimentation rate (ESR) of 25 mm/ hour. A repeat peripheral blood smear (PBS) showed pancytopenia with reticulocyte count increased to 1.3% but still within normal limits. Bone marrow biopsy was advised again.

**Table 2:** Investigation for autoimmune connective disorder.

Investigations parameters	Values
Antiphospholipid antibody IgG/IgM (U/ml)	2.4/4.0
Anti cardiolipin antibody IgG (^*^GPL U/ml)/IgM (||MPL U/ml)	2.5/2.3
ANA	1.07
Direct coombs test	Negative
Haemoglobin in urine (gm/dl)	0.1
ESR (mm/hour)	25

* GPL: G Phospholipid, ||MPL: M Phospholipid.

On day 22, the bone marrow biopsy report showed microcytic hypochromic red blood cell (RBC) with moderate anisopoikilocytosis, leucopenia, thrombocytopenia with mildly hypercellular marrow with the presence of erythroid hyperplasia. The direct coombs test was negative. The patient had hemoglobinuria of 0.1 g/dl.

The patient was advised for a polymerase chain reaction (PCR) assay for the mutation in JAK2 V617F to rule out myelodysplastic syndrome, which came out to be negative.

The patient was suspected to have paroxysmal nocturnal hemoglobinuria because the patient had hemolysis features with indirect hyperbilirubinemia, chronic iron deficiency anemia, and hemoglobinuria, raised ESR, pancytopenia with erythroid hyperplasia of marrow, and thrombosis of a large vessel that is the portal vein. The normal LDH and reticulocyte count could be explained by aplastic anemia overlap. PNH also explained the cause of stroke due to arterial thrombus in this patient.

We advised the patient for the PNH clone flow cytometry test, but due to the unavailability of the test in Nepal, a nationwide lockdown due to the corona virus pandemic, and the poor financial status of the patient, the test could not be performed. On the 24^th^ day (5^th^ May 2020) of admission, we referred the patient with TAB IFOL XT (ferrous ascorbate and folic acid) 1 capsule per oral twice a day, TAB LIPICURE (atorvastatin) 20 mg capsule per oral once a day, TAB MINIL (propranolol) 20 mg per oral twice a day to a hematologist in another hospital for further management.

## DISCUSSION

Cerebrovascular complications of paroxysmal nocturnal hemoglobinuria are frequently reported to be of venous origin, but incidences of arterial occlusions also occur.^6^ So, the likelihood of PNH should be considered in all patients with thrombotic events accompanied by pancytopenia and hemoglobinuria.^7^ Manifestation of anemia during the ischemic event supports the hypothesis of arterial thrombus formation as part of the hemolytic process.^8^

There are three typical complications in patients with PNH which was also evident in our case: hemolysis, evidenced by hemoglobinuria, indirect hyperbilirubinemia, and iron deficiency anemia (IDA); widespread thromboses, substantiated by stroke and extrahepatic venous obstruction; and hematopoietic deficiency, supported by pancytopenia.^9^

PNH is also strongly associated with aplastic anemia, and GPI deficient PNH cells can be detected in greater than 50% of patients with AA. Classical PNH is associated with up to 10-fold elevation in LDH levels, increased reticulocyte count, and moderate thrombocytopenia. Our patient showed moderate to severe thrombocytopenia, no elevation of LDH level, and a normal reticulocyte count, which points toward PNH-AA overlap in the patient.^5^

Bone marrow biopsy showed only slightly hypercellular marrow with no dysplastic cells ruling myelodysplastic syndrome. PCR for JAK2V167F mutation was negative, ruling out myeloproliferative disorder.

Cerebrovascular accidents pertaining to arterial thromboses with pancytopenia, systemic venous thromboses, and hemolysis features were suggestive of PNH but still pose a diagnostic difficulty in a resource-limited country like ours with a lack of testing facility, high cost and poor patients, making its identification and treatment more difficult. The effective treatment is eculizumab, a monoclonal antibody that inhibits membrane attack complex (MAC) formation and intravascular hemolysis. It has been shown to decrease anemia, fatigue, transfusion requirements, renal impairment, pulmonary hypertension, and risk of severe thromboembolic events, ultimately resulting in improving quality of life and survival.^10^ However, the drug isn't available in Nepal and is very expensive for an average Nepali to afford hence, limiting us to symptomatic treatment.

Awareness of variable clinical courses, different PNH variants, diagnostic measures, and overlap syndromes is thus imperative for diagnosis and optimal patient care.
